# Characterizing the gut microbiome of diarrheal mink under farmed conditions: A metagenomic analysis

**DOI:** 10.1371/journal.pone.0312821

**Published:** 2024-10-30

**Authors:** Shuo Liu, Jianwei Ren, Jiyuan Li, Detao Yu, Hang Xu, Fang He, Nianfeng Li, Ling Zou, Zhi Cao, Jianxin Wen

**Affiliations:** College of Veterinary Medicine, Qingdao Agricultural University, Qingdao, Shandong, China; Federal Medical Centre Abeokuta, NIGERIA

## Abstract

This study aimed to comprehensively characterize the gut microbiota in diarrheal mink. We conducted Shotgun metagenomic sequencing on samples from five groups of diarrheal mink and five groups of healthy mink. The microbiota α-diversity and Kyoto Encyclopedia of Genes and Genomes (KEGG) orthology did not show significant differences between the groups. However, significant differences were observed in microbiota β-diversity and the function of carbohydrate-active enzymes (CAZymes) between diarrheal and healthy mink. Specifically, The relative abundance of *Firmicutes* was lower, whereas that of *Bacteroidetes* was higher in diarrheal mink. *Fusobacteria* were enriched as invasive bacteria in the gut of diarrheal mink compared with healthy mink. In addition, *Escherichia albertii* was identified as a new bacterium in diarrheal mink. Regarding functions, nicotinate and nicotinamide metabolism and glycoside hydrolases 2 (GH2) family were the enhanced KEGG orthology and CAZymes in diarrheal mink. Furthermore, the diversity and number of antibiotic-resistant genes were significantly higher in the diarrheal mink group than in the healthy group. These findings enhance our understanding of the gut microbiota of adult mink and may lead to new approaches to the diagnosis and treatment of mink diarrhea.

## Introduction

The mink, being a highly prized fur-bearing animal, possesses a substantial breeding foundation in the regions of North and Northeast China [1]. However, diarrhea consistently poses a considerable challenge for mink breeders and the fur industry throughout the breeding process. Various factors such as dietary patterns, environmental conditions, climatic variations, and pathogenic agents encompassing bacteria, viruses, and parasites can collectively contribute to the onset of acute diarrhea in mink [2]. Therefore, determining the causative factors of diarrhea is difficult, and the indiscriminate use of medication without a clear aetiology may prolong the condition or even result in death, causing more severe economic losses.

The gut microbiota is intricately linked to the physiological processes of the host. Alterations in gut microbiota composition can disrupt the balance of the host’s gastrointestinal ecosystem, thereby affecting normal physiological functions [3]. In recent years, rapid advancements in next-generation sequencing technology have facilitated the comprehensive characterization of diverse facets of microbial communities [4]. Next-generation sequencing has been applied for the detection of gut microbiota in diarrheal mink, however, most studies have focused on pre-weaning diarrhea with few investigations on diarrhea in adult mink.

The present study used metagenomic shotgun sequencing technology to comprehensively investigate the gut microbiota of adult mink with diarrhea, aiming to understand the composition and function of intestinal flora and the distribution of antibiotic-resistance genes in mink with diarrhea. This research establishes a scientific foundation for preventing and treating related diseases, thereby contributing to safeguarding the well-being of humans and animals.

## Materials and methods

### Animals and samples

Five groups of one-year-old healthy white female mink (NM group) and five groups of one-year-old diarrheal white female mink (DM group) were selected from Jiaozhou Haibao Mink Breeding Cooperative, Qingdao, China. The mink were fed a mixture of chicken frame, duck liver, cod, wheat flour, and vitamin supplements. We collected fecal samples from the mink 3 days after observing diarrhea, with the samples being collected 6 h after feeding. None of the mink received antibiotic treatment before fecal collection. Fresh fecal samples were collected using autoclaved 20-mL sampling tubes and transported to the laboratory via a dry-ice cold chain before being stored at −80°C.

### DNA separation, quality inspection, library preparation and metagenomic sequencing

Genomic DNA was extracted from mink feces using the Soil DNA/RNA Extraction Kit (D5625-01, OMEGA Bio-Tek), according to the manufacturer’s instructions. The concentration of extracted DNA was measured using an ultraviolet (UV) spectrophotometer (NanDrop2000), while the quality of the DNA was assessed through 1% agarose gel electrophoresis. Genomic library construction was conducted according to the standardized Illumina TruSeq DNA library preparation protocol. Subsequent sequencing was performed by Shanghai Parsenalbio Biotechnology Co., Ltd. on the Illumina NovaSeq 6000 platform using Paired-end, 2×150 bp mode, generating raw data in the FASTQ format.

### Bioinformatics analysis

Cutadapt (v1.17) was used for identifying and trimming potential splice sequences at the 3′-end, requiring a minimum matching length of 3 bp to the splice sequence and allowing a maximum base mismatch rate of 20%. After removing the 3′-end splice sequences, we performed quality control using fastp (v0.20.0) with a sliding window size of 5 bp, ensuring that the average sequencing accuracy of bases was greater than 99%. Next, using MEGAHIT [5] (parameter set to—k_list 33, 55, 77, 99, 127), The single sample was first spliced, and then the unmapped reads were merged and spliced again to complete the sequence assembly. Only contigs with a length of at least 300 bp were retained. Subsequently, the quality screened and valid sequences were aligned with contigs in their respective samples by minimap2 with default parameters, any sequences that not be aligned were merged and reassembled again. Finally, all contigs sequences were set and merged. The merged contigs sequence set was de-redundant with a similarity threshold of 95% and alignment coverage of 90% using the linclust mode of MMseqs2 software. Open reading frame (ORF) was achieved using MetaGeneMark software (http://exon.gatech.edu/GeneMark/) [6]. Clean data were compared to the Comprehensive Antibiotic Resistance Database (CARD,v3.2.6) [7], and DIAMOND (v2.0.11.149) [8] (with E-value ≤ 1e−5, similarity ≥ 80%, and coverage ≥ 80%) was used for fast comparison to identify antibiotic resistance genes (ARGs) in samples. The results were then annotated by contrast using the annotation information of the CARD data. We used the cluster module of MMseqs tool to remove redundancy based on 95% similarity and 90% coverage of the alignment region, obtaining the non-redundant protein set. Subsequently, the taxonomy module of MMseqs tool was used to align the non-redundant proteins with the National Center for Biotechnology Information (NCBI) NR database (v2021.10.11), and the species information with the highest alignment score was selected as the source of the species information of the protein gene sequence under the sensitivity parameter setting of 5.7 and lca-mode setting of 4.

For functional annotation of protein sequences, the predicted protein set was compared with the protein database (v2020.10.20) provided by KOBAS software [9] and dbCAN (database for automated carbohydrate-active enzyme annotation) [10] reference protein library (v2021-09_24) was used for comparison, MMseqs2 was used for alignment, the sensitivity parameter was set to 5.7, and Kyoto Encyclopedia of Genes and Genomes (KEGG) orthology (KO) and carbohydrate-active enzymes (CAZymes) information was obtained by selecting the reference protein data with the highest alignment score to determine the alignment results.

### Statistical analysis

QIIME software [11] was used to calculate α-diversity indices (Simpson, Chao1, ACE, Shannon). Specaccum species accumulation curves were plotted using R software to assess sample adequacy and estimate community richness. β-diversity of the samples was assessed using the Bray-Curtis distance and the Adonis test was conducted using QIIME software, with 999 permutation tests conducted to assess the statistical significance of intergroup differences [12].

Species Venn diagrams were used to demonstrate inter-group species differences. Linear discriminant analysis Effect Size (LEfSe) analysis was used for identifying differential species and functional groups [13]. The Shannon index and abundance indices were calculated using the relative abundance spectra of ARGs. Principal coordinate analysis (PCoA) was performed using the Bray-Curtis distance, and the significance of differences between groups was determined using permutation multivariate analysis of variance (PERMANOVA). The Wilcoxon rank-sum test was used to assess whether there were significant differences between the diversity indices and relative abundances of taxa, and ARGs between the different groups. All other visualizations were performed in R software.

## Results

### Composition and diversity of gut microbiota

**Fig 1:**
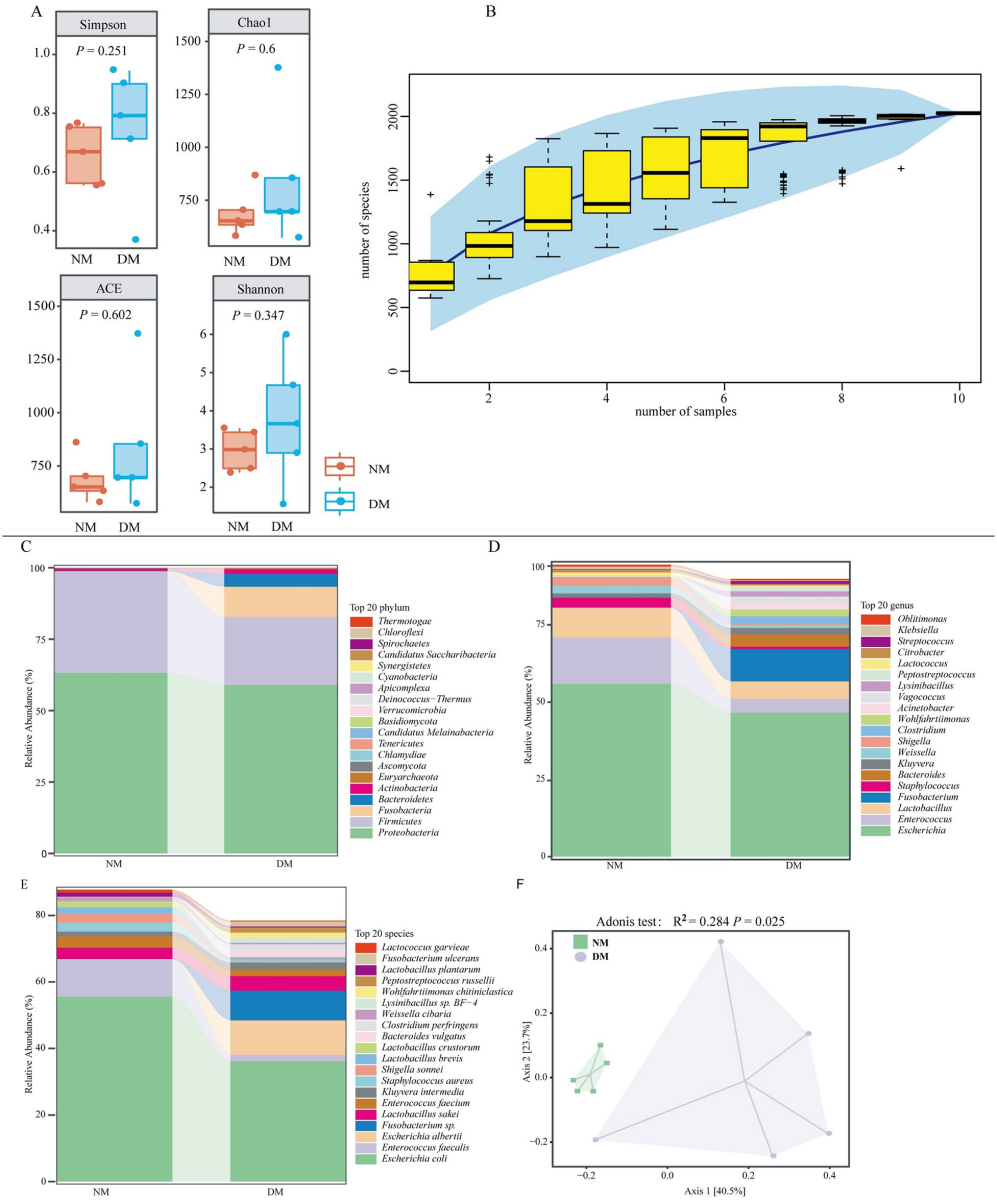
Composition and diversity of gut microbiota of diarrheal and healthy mink. (A) α-diversity: Simpson, Chao1, ACE and Shannon. (B) A plot of Specaccum species accumulation at the species level, the abscissa represents the sample size, the ordinate represents the number of species examined, and the blue shading reflects the confidence intervals of the curves. (C–E) Taxonomic composition of gut microbiota at phylum, genus and species levels between the NM and DM groups. (F) Principal Coordinate Analysis (PCoA) was used to show the β-diversity Adonis test: R^2^ = 0.284 *P* < 0.05. DM, diarrheal mink; NM, healthy mink.

α-diversity was determined at the minimum sequencing depth using four indices: Simpson, Chao1, ACE, and Shannon (Fig 1A, S1 Table). No statistical significance differences (*P* > 0.05) were observed in these indices. As the sample size increased, the number of detectable species increased, plateauing at a sample size of 10, indicating that this sample size sufficiently captures community species composition (Fig 1B). PCoA revealed that the gut microbiota in the NM group tended to cluster, whereas the DM group showed more dispersion. In addition, there was a significant distance between the NM and DM groups. Axis1 and Axis2 contributed 40.5% and 23.7% of the variance, respectively. The Adonis test confirmed a significant difference between NM and DM groups (R^2^ = 0.284 *P <* 0.05) (Fig 1F, S2 Table). Sequence alignment of non-redundant protein sequences against the NCBI NR database identified 20 phyla, 36 classes, 562 genera, and 2024 species. We further explored the top 20 genera, families, and species. At the phylum level, the NM group was dominated by *Proteobacteria* (63.2%), *Firmicutes* (35.6%), and *Actinobacteria* (0.8%), and the DM group was dominated by *Proteobacteria* (59.0%), *Firmicutes* (23.9%), *Bacteroidetes* (10.5%), and *Actinobacteria* (4.5%) (Fig 1C). In the NM group, the most abundant genera were *Escherichia* (55.9%), *Enterococcus* (15.0%), and *Lactobacillus* (9.6%), whereas, in the DM group, the most abundant genera were *Escherichia* (46.6%), *Lactobacillus* (5.6%), and *Clostridium* (10.5%) (Fig 1D). The predominant species in the NM group were *Escherichia coli* (55.6%), *Enterococcus faecalis* (11.3%), and *Enterococcus faecium* (3.4%), while in the DM group, the dominant species were *Escherichia coli* (36.2%), *Escherichia albertii* (10.3%), and *Fusobacterium sp*. (8.9%) (Fig 1E).

### Differences in gut microbiota

**Fig 2:**
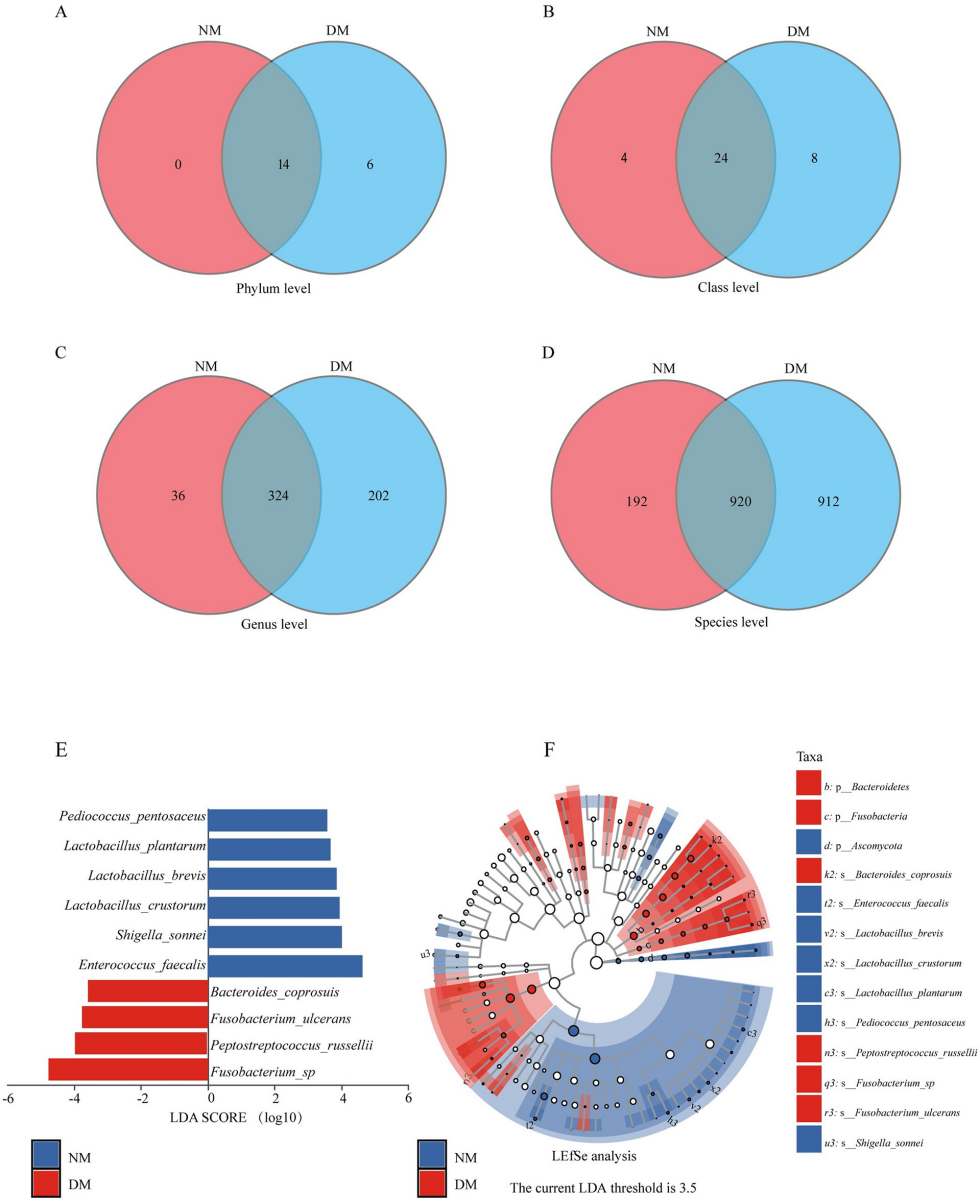
Taxonomic variations between gut microbiota of diarrheal and healthy mink. (A–D) Venn diagrams of phylum, class, genus and species level. (E) Histogram of the LDA value distribution of significantly different species showing the significantly enriched species and their importance in each group. (F) Cladogram showing the taxonomic hierarchy of the marker species in each group of samples. DM, diarrheal mink; NM, healthy mink.

The Venn diagrams showed that 14 phyla were shared between the two groups of mink, while the DM group had 6 unique phyla (Fig 2A). At the class level, 24 classes were shared between the two groups, while the NM had 4, and the DM had 8 unique classes (Fig 2B). At the genus level, 324 shared genera were shared, with NM and DM groups having 36 and 206 unique genera, respectively (Fig 2C). At the species level, 920 species were shared, with the NM and DM groups possessing 192 and 912 unique species, respectively (Fig 2D). LEfSe analysis (linear discriminant analysis (LDA) score ≥ 3.5, P < 0.05) revealed that significant differential taxa in the DM group were primarily within *Fusobacteria* and *Bacteroidetes*, while the NM group showed predominant taxa in *Ascomycota*. The LDA score chart revealed significant differences in the microbiota composition: *Enterococcus faecalis*, *Shigella sonnei*, *Lactobacillus crustorum*, *Lactobacillus brevis*, *Lactobacillus plantarum*, and *Pediococcus pentosaceus* were enriched in the NM group, whereas *Bacteroides coprosuis*, *Peptostreptococcus russellii*, *Fusobacterium ulcerans* and others were enriched in the DM group (Fig 2E and 2F).

### Functional profiles of gut microbiota

**Fig 3:**
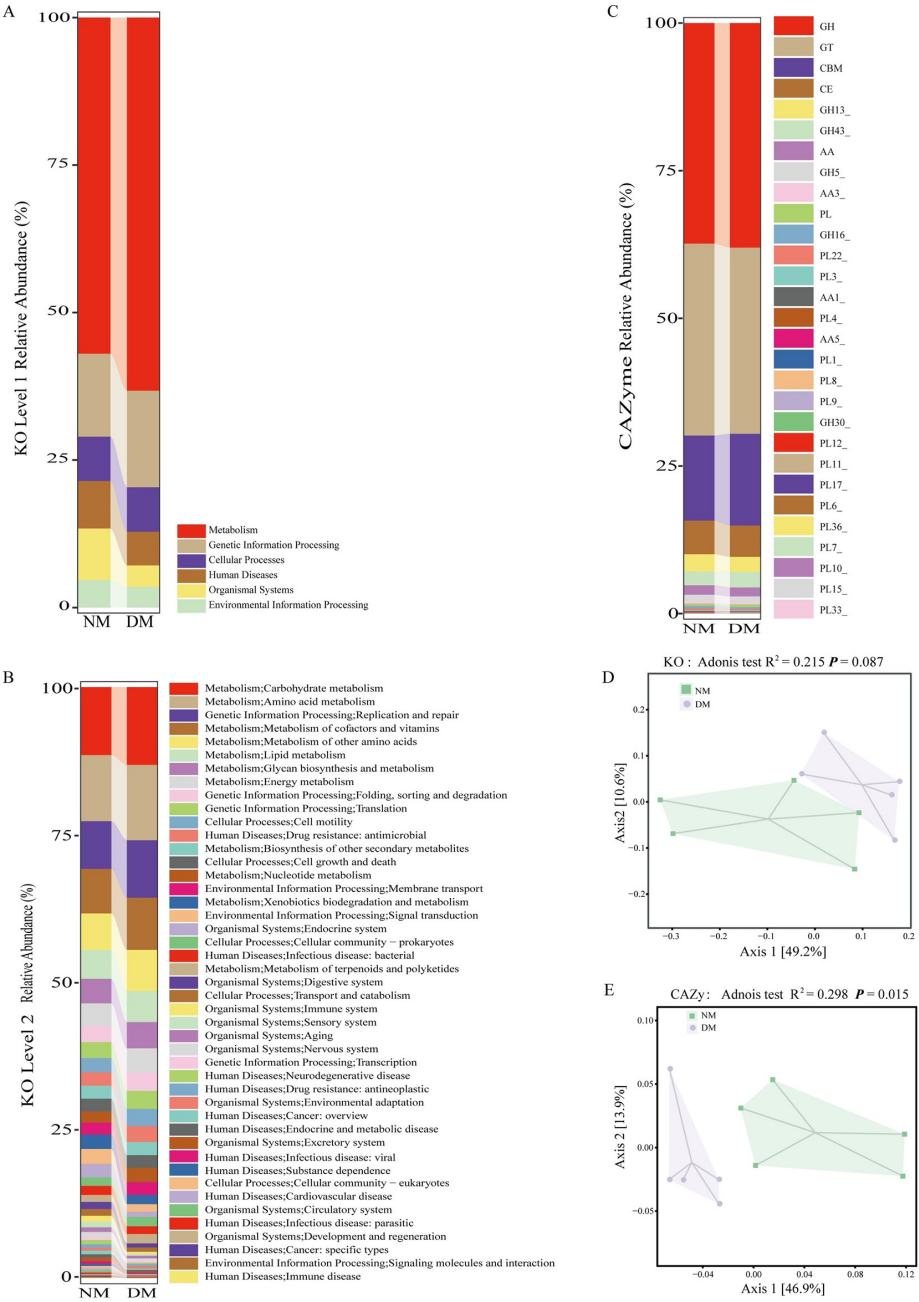
Functional profiles of gut microbiota of diarrheal and healthy mink. (A, B) KEGG orthology (KO) relative abundance in the gut microbiota of diarrheal mink and healthy mink groups. (C) Carbohydrate-active enzymes (CAZymes) relative abundance in the gut microbiota of NM and DM groups.Note: Glycoside Hydrolase (GH), Glycosyltransferase (GT), Carbohydrate-Binding Module (CBM), Carbohydrate Esterases (CE), Auxiliary Activities (AA), Polysaccharide Lyase(PL). (D, E) Differences in KO and CAZymes between NM and DM groups were based on principal coordinate analysis (PCoA) of the Bray-Curtis distance and Adonis test.

The annotation results according to the KO database showed that at KO level 1, the relative abundance of Metabolism and Genetic Information Processing was higher in the DM group, while Organismal Systems and Environmental Information Processing decreased were lower compared with the NM group (Fig 3A). At KO level 2, the top five functions by relative abundance in the DM and NM groups were Carbohydrate metabolism, Amino acid metabolism, Replication and repair, Metabolism of cofactors and vitamins and Metabolism of other amino acids (Fig 3B). The annotation results according to the CAZymes database indicated that Glycoside Hydrolase (GH), Glycosyltransferase (GT), Carbohydrate-Binding Module (CBM), Carbohydrate Esterases (CE) and Glycoside Hydrolase 13 (GH13) were the five most abundant CAZyme families in both groups (Fig 3C). PCoA and Adonis test showed that the DM group were clustered together, while the NM group were relatively dispersed. Axis1 and Axis2 contributed 49.2% and 10.6% to the KO variance, and 46.9% and 13.9% to the CAZyme variance, respectively. There was no statistically significant difference in KO between NM and DM groups, but a significant difference was observed in CAZyme (KO: R^2^ = 0.215 *P* > 0.05, CAZyme: R^2^ = 0.298 *P* < 0.05) (Fig 3D and 3E, S3–S6 Tables).

### Functional differences of gut microbiota

**Fig 4:**
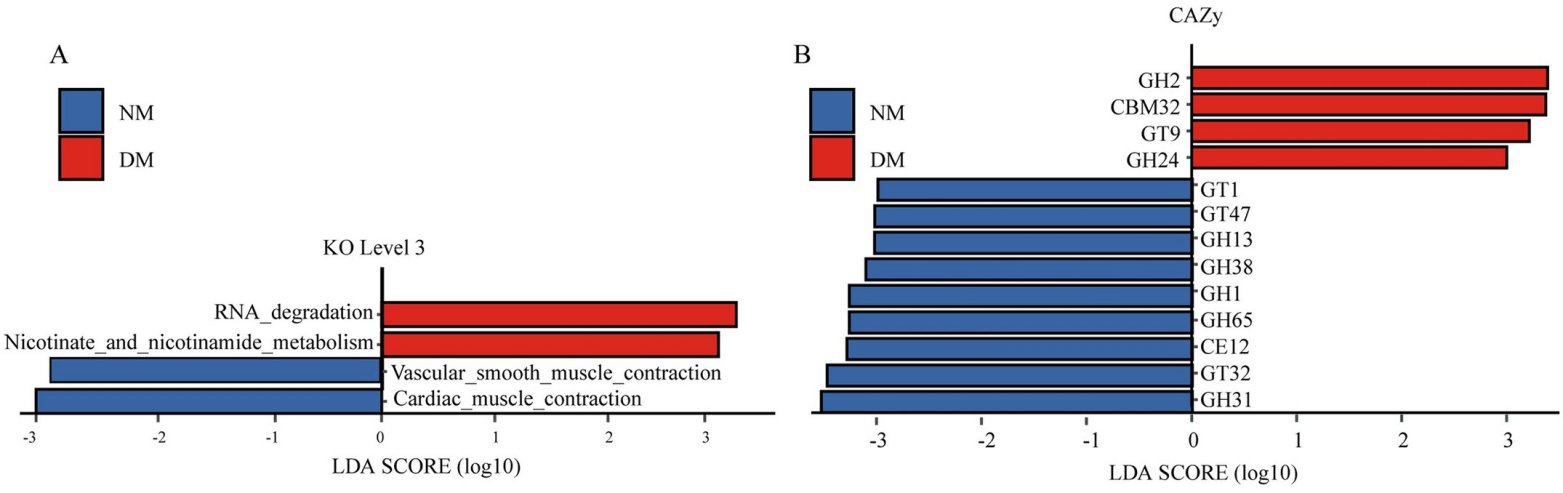
Functional differences of gut microbiota of diarrheal and healthy mink. (A, B) Functional abundance tables of KEGG and CAZyme databases were analyzed separately by Linear discriminant analysis (LDA) Effect Size (LEfSe) local analysis software. The ordinate is the taxon with significant differences between groups, and the abscissa is a bar chart to visually show the log score value of LDA analysis for each taxon.

LEfSe analysis (LDA scores ≥ 2.9 *P* <0.05) identified 35 KO, and 84 CAZymes, that were significantly different between the two groups of mink. In the DM group, the functions of RNA degradation, and Nicotinate and nicotinamide metabolism were enriched, while Cardiac muscle contraction and Vascular smooth muscle contraction were increased in the NM group (Fig 4A). For CAZyme functions GH2, CBM32, GT9 and GH24 were enhanced in the NM group, whereas GT1, GT47, GH38, GH1, GH65, CE12, GT32, and GH31 were enhanced in the DM group (Fig 4B).

### Antibiotic-resistance genes (ARGs) of the gut microbiome

**Fig 5:**
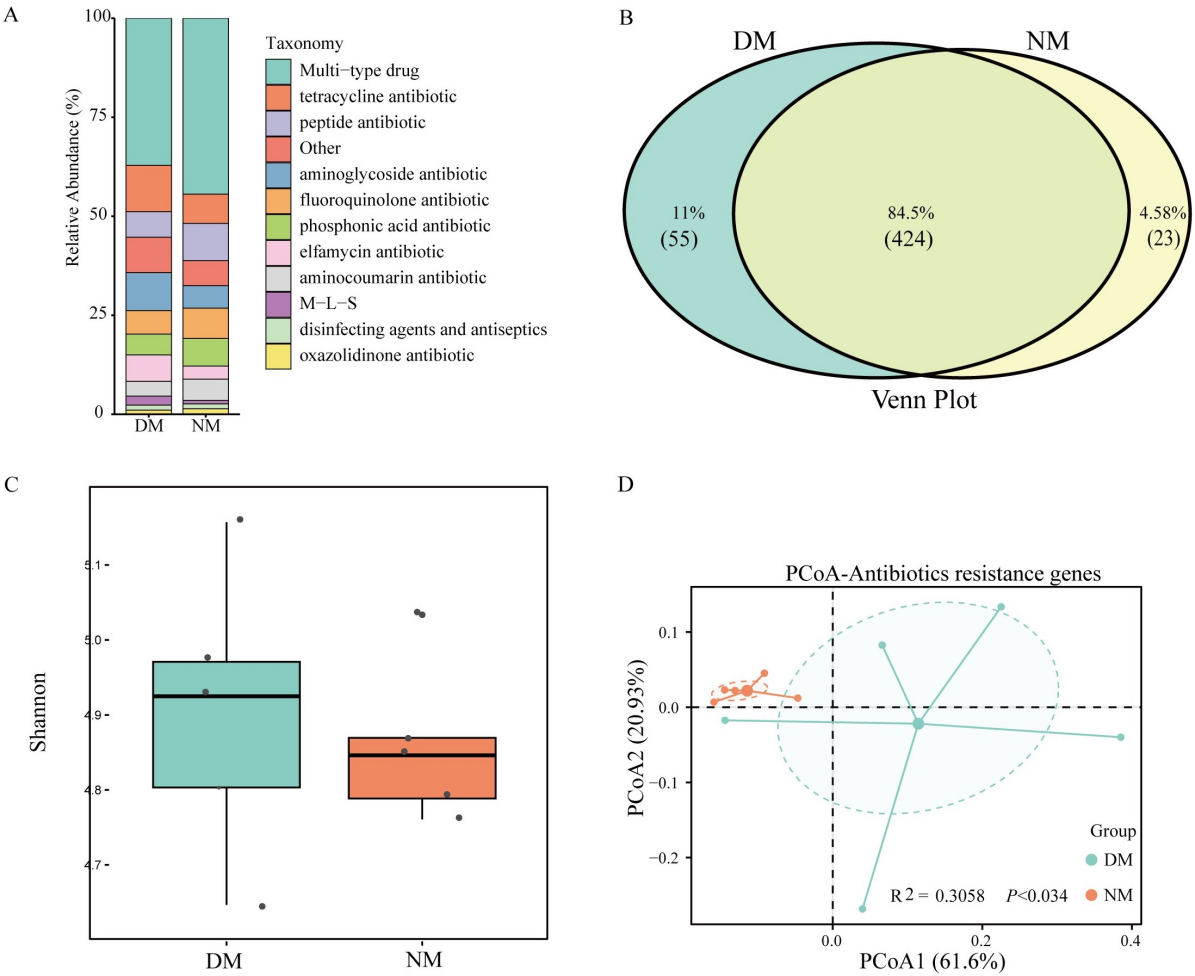
Antibiotic-resistance genes (ARGs) of the gut microbiome of diarrheal and healthy mink. (A) Relative abundance of ARGs in the gut microbiota of diarrheal mink (DM) and healthy mink (NM) groups. (B) Venn diagram showing ARGs that are common and unique to the NM group and DM groups. (C) Box plots generated using the Shannon index and showing differences in ARGs, between DM and NM groups. (D) The differences in ARGs between NM and DM groups were based on PCoA of Bray—Curtis distance and permutation multivariate analysis of variance (PERMANOVA).

Through matching with CARD database, 4496 resistance genes were detected in this study (S7 Table), which were categorized into 12 ARGs: multidrug, tetracycline, peptide antibiotic, aminoglycoside antibiotic, fluoroquinolone antibiotic, phosphonic acid antibiotic, elfamycin antibiotic, aminocoumarin antibiotic, macrolide—lincosamide—streptogramin antibiotics (M–L–S), disinfecting agents and antiseptics, oxazolidinone antibiotic, and others. Multidrug resistance had the highest relative abundance in both groups of mink (dirrheal and healthy), followed by tetracycline antibiotics. (Fig 5A). A total of 502 subtypes of ARGs were detected in both groups, including 424 core subtypes, 55 unique subtypes in the DM group and 23 unique subtypes in the NM group (Fig 5B). The Shannon index box plot and PCoA revealed that the diversity and abundance of ARGs in the DM group were significantly higher compared with the NM group (PERMANOVA, R^2^ = 0.3058, *P* < 0.034) (Fig 5C and 5D).

## Discussion

The gut microbiota is an ecosystem intricately linked to the host, playing essential roles in nutrition, immune system modulation, and host defence. These functions of the gut microbiota are crucial for establishing protective barriers and the maintenance of overall host health [14, 15]. However, the delicate homeostatic balance of the gut microbiota can be easily disturbed by exogenous microorganisms, thereby disrupting homeostasis and increasing susceptibility to diarrhea and other diseases [16]. Diarrhea is a common condition in humans and animals and generally results from an imbalance in intestinal flora caused by pathogenic infections (e.g., fungi, bacteria, and viruses), ultimately resulting in the manifestation of diarrhea [17]. Although numerous studies have investigated mink diarrhea, most have focused on pre-weaning diarrhea [18], with limited attention given to alterations in the gut microbiota of adult mink. In this study, we utilized metagenomic sequencing technology to comprehensively examine the gut microbiota of adult mink with diarrhea, providing novel insights for managing diarrheal disease in animals.

We observed no significant differences in α-diversity between the NM and DM groups. However, a statistically significant difference in β-diversity was detected in the gut microbiota of the mink groups. Furthermore, Venn diagrams showed that more microbial taxa were annotated from phylum to species in the DM group, indicating structural changes in the microbiota associated with diarrhea. Specifically, at the phylum level, the relative abundance of *Firmicutes* decreased, and *Bacteroidetes* increased in diarrheal mink (NM vs DM 0.1% vs 4.5%), which is consistent with findings in human colitis studies [19]. However, differences in study subjects, mean that this observation cannot be considered a definitive maker for mink diarrhea. Zhao et al. [20] reported the widespread presence of *Fusobacteria* in the mink gut, whereas our results revealed that Fusobacteria (DM vs NM 10.5% vs 0%) were identified as invasive bacteria in the gut of diarrheal minks. Geographical region and dietary structure may be the main reasons for the differences in results between studies.

The present investigation revealed that *Enterococcus faecium* and *Enterococcus faecalis* were the predominant species within the genus *Enterococcus* in minks. These bacteria, as symbiotic residents of a host’s gut, are instrumental in nutrient metabolism, improving the composition of the gut microbiota, maintaining intestinal homeostasis, and preventing diarrhea [21–23]. However, the relative abundance of *Enterococcus* (NM vs DM 15% vs 4.4%) and *Lactobacillus* (NM vs DM 9.6% vs 5.6%) exhibited a significant decrease in diarrheal minks. This phenomenon may be related to the infection of *Fusobacterium* (*Fusobacterium ulcerans* (1.5%), *Fusobacterium sp* (8.9%) and other pathogenic bacteria. Birch et al. [24] demonstrated a decrease in the relative abundance of *Staphylococcus* in the diarrhea group during their investigation of pre-weaning diarrhea, which aligns with our findings and suggests that *Staphylococcus* may not be implicated as a causative agent for diarrhea. Contrary to the findings of Williams et al. [25], at the species level, we observed the presence of *Shigella sonnei* in both groups of mink. *Shigella sonnei* is believed to have undergone evolutionary divergence from *Escherichia coli* and serves as the primary etiological agent of bacillary dysentery in human and animal hosts [26, 27]. However, the relative abundance of *Shigella sonnei* in the DM group in the present study was minimal and significantly lower than that observed in the NM group (NM vs DM 2.7% vs 0.4%). Consequently, it is reasonable to hypothesise that *Shigella sonnei* does not exhibit pathogenicity toward mink diarrhea. A distinct species known as *Escherichia albertii* was previously identified in the intestinal tract of diarrheal minks [28] and exhibited phenotypical and genetic similarities to *Escherichia coli*. *Escherichia albertii* commonly carries the *eae* virulence gene, with a subset of strains possessing highly infectious *Shigella toxin 2* (*stx2a*, *stx2f*) genes, resulting in the manifestation of symptoms such as watery diarrhea, fever, and abdominal pain in the host [29, 30]. Hinenoya et al. [31] isolated *Escherichia albertii* from the feces of wild mink, foxes, and rabbits in Okayama Prefecture, Japan, suggesting that mink can be infected with *Escherichia albertii* through food-borne transmission. Qun Li et al. [32] demonstrated that *Escherichia albertii* can be transmitted through raw meat, such as chicken and duck. Given the carnivorous nature of mink, which predominantly consumes minced poultry meat during feeding, we postulated that this could be one potential route for mink infection.

Our study revealed that the DM group exhibited a higher relative abundance of Metabolism and Genetic Information Processing at KO level 1, compared with the NM group, while the relative abundance of Organismal Systems and Environmental Information. These findings align closely with a previous investigation on irritable bowel syndrome [33]. In the present study, the top five functions with relatively higher abundance in the NM group are Carbohydrate metabolism, Amino acid metabolism, Replication and repair, Metabolism of cofactors and vitamins, as well as Metabolism of other amino acids. All five functions also have a higher relative abundance in the DM group compared with the NM group. We believe that this is an adaptive response of intestinal flora to maintain host metabolic homeostasis [34]. At the KO level 3, we observed that the DM group exhibited prominent differential functions related to RNA degradation, as well as Nicotinate and nicotinamide metabolism, whereas the NM group showed enhanced Vascular smooth muscle contraction and myocardial contraction. We suggest that the increased nicotinate metabolism in the DM was a response to the imbalance in gut microbiota and the reliance of the intestine on the anti-inflammatory properties of nicotinate [35].

The gut microbiota also contains a substantial repertoire of CAZymes that are crucial for the assembly or degradation of oligosaccharides and polysaccharides to fuel vital metabolic processes in mink. In the present study, we found that GHs and GTs are the predominant CAZymes in the mink gut. GH2 and GT1 were the most abundant differential CAZyme functions in the intestine of the DM and NM groups, respectively. β-galactosidases is a key enzyme in the GH2 family that naturally catalyzes β-galactosidic linkages [36], while enzymes from the GT1 family demonstrate exceptional glycosylation proficiency towards glycolipids, flavonoids, macrolides, and other compounds [37]. These findings suggest that diarrheal mink have a reduced capacity for glycosylation of small molecules and an increased ability to degrade polysaccharides or oligosaccharides. PCoA and Adonis tests showed significant differences in CAZyme functions between NM and DM groups, which are closely related to alterations in the gut microbiota structure [38].

Exploration of mink intestinal ARGs revealed that the NM and DM groups contained 502 ARG subtypes and were resistant to 12 types of ARGs. The most common of these are genes that confer resistance to multidrugs, tetracyclines and aminoglycosides. The source of resistance is related to the mink’s diet, disease treatment, and living environment of mink [39]. For caged mink, the high frequency of contact with humans is one reason why these animals become hosts of ARGs [40]. Chicken, duck and fish, as a daily food source for mink, are another route through which mink can obtain ARGs [41,42]. We observed that the diversity and number of ARGs in the DM group were higher than those in the NM group. Given that all mink were subjected to identical feeding conditions in our study, we infer that this result is closely associated with changes in gut microbiota structure. Since bacteria carry ARGs, shifts in bacteria abundance can directly impact ARG levels [43].

## Conclusion

This study used metagenomic shotgun sequencing to analyze the differences in the structure and function of the gut microbiota and the distribution of ARGs between diarrhea mink and healthy mink. *Fusobacterium ulcerans*, *Fusobacterium sp*., and *Escherichia albertii* were enriched as invasive bacteria in the intestine of diarrheal mink. Concurrently, there were significant differences in the function of CAZymes between the two groups of mink. In addition, the diversity and number of ARGs were significantly higher in diarrheal mink compared with those in healthy mink. Consequently, we conclude that *Fusobacterium* and *Escherichia albertii* play a crucial role in mink diarrhea. In addition, alteration in microbiota structure may lead to changes in CAZyme functions and ARGs abundance in the gut. This study, which is the first to comprehensively analyze the intestinal flora of adult mink with diarrhea, provides new therapeutic targets for the treatment of diarrhea in mink and novel insights for managing feeding and antibiotic use in these animals.

## Supporting information

S1 Table Alpha-diversity. (XLSX)

S2 Table Beta-diversity Adonis test. (XLSX)

S3 Table KO_PCoA summary. (XLSX)

S4 Table CAZyme_PCoA summary. (XLSX)

S5 Table KO Adonis test. (XLSX)

S6 Table CAZyme Adonis test. (XLSX)

S7 Table ARGs summary. (XLSX)
